# Ncf1/Igfbp2 Signaling Regulates Airway Submucosal Gland Progenitor Fitness and Ionocyte Specification

**DOI:** 10.64898/2026.10.13.758956

**Published:** 2026-10-15

**Authors:** Javahir Hajibabazade, Kyhler D. Campbell, Oluwadarasimi Atanda, Grace Fritz, Charles A. Yeaman, Weam S. Shahin, John F. Engelhardt

## Abstract

**Background:**

Airway submucosal gland progenitors (SMGPs) serve as reserve stem cells for epithelial regeneration, yet mechanisms governing their long-term maintenance remain poorly understood. Here, we identify Ncf1/Igfbp2 signaling as a regulator of mouse SMGP fitness and epithelial lineage specification.

**Methods:**

Mouse SMGPs with genetic disruption of *Ncf1* or *Igfbp2* were serially passaged to assess progenitor proliferation, organoid-forming capacity, epithelial marker expression, DNA damage, DNA repair responses, and telomere length. Differentiated epithelia were evaluated for Cftr-mediated anion transport and pulmonary ionocyte specification in vitro and in vivo.

**Results:**

Genetic disruption of *Ncf1* or *Igfbp2* preserved SMGP identity, enhanced proliferative and organoid-forming capacity, and reduced DNA damage accumulation through activation of DNA repair programs. Epithelia derived from *Ncf1* ^−/−^ and *Igfbp2* ^−/−^ SMGPs maintained higher Cftr-mediated anion transport at late passage and exhibited enhanced pulmonary ionocyte specification in vitro and in vivo. These effects occurred independently of telomere shortening, implicating altered stress responses and DNA damage handling rather than replicative limits.

**Conclusions:**

Together, our findings identify Ncf1/Igfbp2 signaling as a regulator of airway stem cell fitness and epithelial maturation, with implications for regenerative therapies in lung diseases such as cystic fibrosis.

